# Dissecting the Function of MADS-Box Transcription Factors in Orchid Reproductive Development

**DOI:** 10.3389/fpls.2019.01474

**Published:** 2019-11-15

**Authors:** Zhi Wei Norman Teo, Wei Zhou, Lisha Shen

**Affiliations:** ^1^Temasek Life Sciences Laboratory, National University of Singapore, Singapore, Singapore; ^2^Department of Biological Sciences, Faculty of Science, National University of Singapore, Singapore, Singapore

**Keywords:** orchid, MADS-box transcription factors, floral transition, floral patterning, development

## Abstract

The orchid family (Orchidaceae) represents the second largest angiosperm family, having over 900 genera and 27,000 species in almost all over the world. Orchids have evolved a myriad of intriguing ways in order to survive extreme weather conditions, acquire nutrients, and attract pollinators for reproduction. The family of MADS-box transcriptional factors have been shown to be involved in the control of many developmental processes and responses to environmental stresses in eukaryotes. Several findings in different orchid species have elucidated that MADS-box genes play critical roles in the orchid growth and development. An in-depth understanding of their ecological adaptation will help to generate more interest among breeders and produce novel varieties for the floriculture industry. In this review, we summarize recent findings of MADS-box transcription factors in regulating various growth and developmental processes in orchids, in particular, the floral transition and floral patterning. We further discuss the prospects for the future directions in light of new genome resources and gene editing technologies that could be applied in orchid research and breeding.

## Introduction

The orchid family (Orchidaceae) is currently the second largest angiosperm family, having over 900 genera and 27,000 species in almost all parts of the world except Antarctica. New genera in the orchid family are being discovered at a rate of around 13 per year for over the past decade (Schuiteman, 2004; Chase et al., 2015). Nevertheless, many wild species are at the brink of extinction because of illegal trading activities (Wijnstekers, 2001; Hossain et al., 2013). Orchids have their own ecological niches through their relationships with mycorrhizal fungi, specialized pollinators and host trees (Fay and Chase, 2009). Of all the orchid species, 70% are epiphytic (growing on trees), 25% are terrestrial (growing on ground), and the remaining 5% are found on various supports such as rocks (Atwood, 1986). Thus, it is important to conserve orchid species through generating awareness and increasing our understanding on the species physiology and diversity (Cribb et al., 2003).

Being a class of valuable ornamental plants with distinct and attractive flowers, orchid is viewed as a high value commodity in the global flower cultivation and landscaping industries. They are also highly sought for as food and traditional medicine (Arditti, 1992; Bulpitt et al., 2007). Besides their great economic values, orchids are also exclusive genetic resources for studying plant developmental processes, including floral transition, floral development, flower pigmentation, and senescence, because of the specialized reproductive structures and the unique strategies for reproduction (Yu and Goh, 2001; Gutiérrez, 2010; Da Silva et al., 2014). However, the orchid research as well as orchid breeding have been challenging due to the long vegetative developmental period before switching to flower development, and technical limitations in transformation and obtaining transgenic lines in various orchid species. Currently, to investigate the function of orchid genes, several methods are being used including the heterologous expression of gene of interest under the strong constitutive CaMV *35S* promoter in *Arabidopsis* (*Arabidopsis thaliana*) or tobacco and virus-induced gene silencing (VIGS). Sometimes, transgenic orchids are also generated to study gene function. So far, the genetic transformation of orchids using the *Agrobacterium*-mediated approach on protocorm-like bodies or rhizomes has been reported in *Cymbidium*, *Oncidium*, *Dendrobium*, *Phalaenopsis*, and other orchids (Belarmino and Mii, 2000; Yu and Goh, 2000; Chai et al., 2002; Chen, 2002; Sjahril and Mii, 2006; Shrestha et al., 2007; Zhang et al., 2010; Ding et al., 2013).

The orchids including *Phalaenopsis*, *Dendrobium*, *Cymbidium*, and *Oncidium* from the Epidendroideae subfamily are used as orchid plant models for research and biotechnology. Most of these orchids are predominantly found to be growing in tropical Asia to Australia with the exception of *Oncidium* in the West (**Table 1**). Orchids are either monopodial or sympodial in their growing habits. Monopodial orchids, such as *Phalaenopsis*, grow as a single erect “stem” with alternating leaves on opposing parts of the center. They store water in their thick leaves and roots but have no pseudobulbs. Sympodial orchids, such as *Dendrobium*, *Cymbidium*, and *Oncidium*, grow from a horizontal stem called rhizome and have pseudobulbs to store water and grow new leaves. After blooming, the plant will resume growth at axillary buds at the base of the previous pseudobulbs.

**Table 1:** Orchid model plants and their growing characteristics.

Genus	Distribution	Branching architecture	Characteristics
*Cymbidium*	From the Himalayan region eastwards to Southeast Asia, China, and Australia	Sympodial	Mostly terrestrial Large, round pseudobulbs (stems) Long thin leaves Thick roots
*Oncidium*	South America, Central America, Mexico, and the West Indies	Sympodial	Mostly epiphytic Presence of column wings Pseudobulbs with one to three leaves Pseudobulbs having several basal bracts at the base
*Dendrobium*	Tropical Asia, islands of the Pacific, New Guinea, and Australia	Sympodial	Mostly epiphytic Generating new stems (pseudobulbs) at the base of the previous year’s stems
*Phalaenopsis*	India, China, Southeast Asia, New Guinea, and Australia	Monopodial	Mostly epiphytic Long and coarse roots Short and leafy stems Flat flowers arranged in a flowering stem that often branches near the end

Recent findings from different orchid species have elucidated that a group of MADS-box transcription factors sharing a greatly conserved N-terminal DNA binding domain (MADS-box) exert important functions in controlling orchid growth and development, in particular, the floral transition and floral patterning. In this review, we discuss the biological roles of these MADS-box proteins and the mechanisms how they contribute to flowering and floral organ formation in orchids. We further elaborate about the prospects for research and development in light of new genome resources and gene editing technologies that could be applied in orchid research and breeding.

## The Mads-Box Protein Family

In orchids and other angiosperms, there is a family of MADS-box transcription factors that have been identified to control many plant developmental processes, including floral transition, floral patterning, as well as male and female gametophyte development (Coen and Meyerowitz, 1991; Weigel and Meyerowitz, 1994; Yu and Goh, 2000; Acri-Nunes-Miranda and Mondragón-Palomino, 2014; Valoroso et al., 2019). The MADS-box family proteins are conserved in nearly all eukaryotes. The MADS-box acronym is derived from the yeast *MINICHROMOSOME MAINTENANCE 1* (*MCM1*) (Passmore et al., 1988), the *Arabidopsis AGAMOUS* (*AG*) (Yanofsky et al., 1990), the *Antirrhinum DEFICIENS* (*DEFA*) (Schwarz-Sommer et al., 1990), and the mammalian *SERUM RESPONSE FACTOR* (*SRF*) (Norman et al., 1988; Gramzow et al., 2010). All identified MADS-box proteins each contain a MADS-box domain of ∼58 amino acid at the N-terminus that binds to a consensus CC[A/T]_6_GG sequence, termed as the “CarG-box” motif (Hayes et al., 1988; Riechmann et al., 1996). Interestingly, flowering plants (angiosperms) have more of these genes (e.g. 107 in *Arabidopsis*; 51 in *Phalaenopsis equestris*) compared to yeast (e.g. 4 in *Saccharomyces cerevisiae*) and mammals (e.g. 5 in *Homo sapiens*) (Becker and Theißen, 2003; Messenguy and Dubois, 2003; Par˘enicová et al., 2003; Cai et al., 2015). The MADS-box family of genes form two major lineages, namely type I of *SRF-*like genes and type II of *MEF2-*like genes, which is resulted from an ancient event of gene duplication prior to the divergence of the kingdoms of plants and animals (Alvarez-Buylla et al., 2000). In plants, type II genes of MADS-box, also called as MIKC-type genes, feature four distinct protein domains arranged from the N-terminal end to C-terminal end. They are the highly conserved DNA binding MADS-box (M) domain, the less-conserved intervening domain (I) for conferring interaction specificity between different MADS-box transcription factors and/or other proteins, the keratin-like coiled-coil (K) domain for conferring protein–protein interactions, and a highly variable C-terminal (C) domain for regulating gene transcription or multimeric protein complexes formations (Shore and Sharrocks, 1995; Theißen et al., 1996; Riechmann and Meyerowitz, 1997; Honma and Goto, 2001; Becker and Theißen, 2003; Hill et al., 2008).

**Figure 1:**
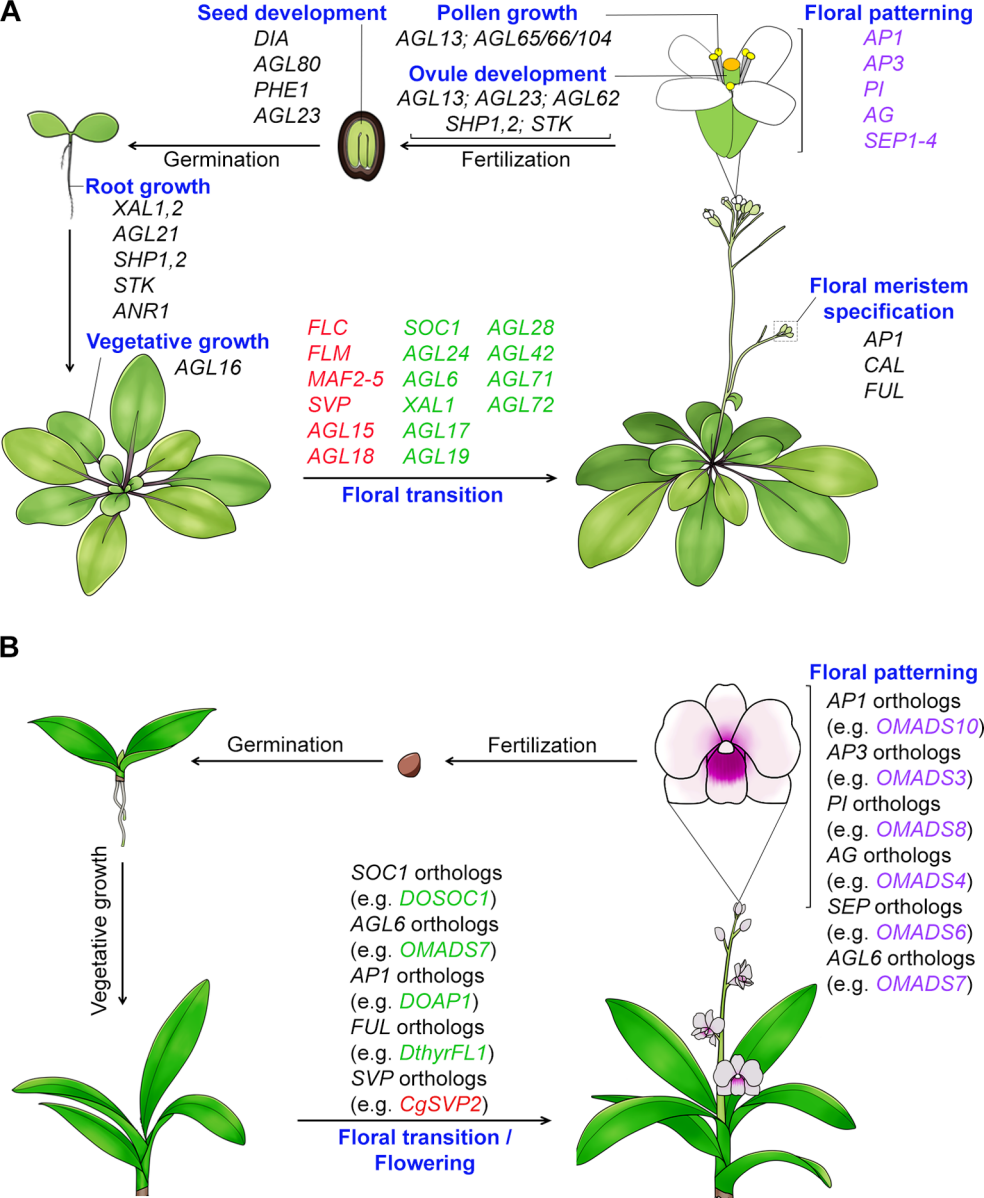
Function of MADS-box proteins in the whole plant life cycle. **(A)** MADS-box genes regulate *Arabidopsis* development throughout its life cycle. Many MADS-box genes mediate the transition to flowering. The flowering time repressor genes, including *FLC* (Michaels and Amasino, 1999), *FLM* (Ratcliffe et al., 2001), *MAF2-5* (Ratcliffe et al., 2003; Gu et al., 2013), *SVP* (Hartmann et al., 2000; Li et al., 2008), and *AGL15/18* (Adamczyk et al., 2007), are shown in red color, whereas the flowering time promoter genes, including *SOC1* (Lee et al., 2000), *AGL24* (Yu et al., 2002), *AGL6* (Yoo et al., 2011), *XAL1/AGL12* (Tapia-Lopez et al., 2008), *AGL17* (Han et al., 2008), *AGL19* (Schonrock et al., 2006), *AGL28* (Yoo et al., 2006), and *AGL42/71/72* (Dorca-Fornell et al., 2011), are shown in green color. All the identified floral organ identity genes except *AP2* encode MADS-box transcription factors. MADS-box genes are also involved in root growth (e.g. *XAL1*, *XAL2*, *AGL21*, *ANR1, SHP1,2*, and *STK*) (Zhang and Forde, 1998; Tapia-Lopez et al., 2008; Moreno-Risueno et al., 2010; Garay-Arroyo et al., 2013; Yu et al., 2014), vegetative growth (e.g. *AGL16*’s function in stomata development) (Kutter et al., 2007), pollen maturation and tube growth (*AGL65/66/104*) (Adamczyk and Fernandez, 2009), ovule development (e.g. *AGL13*, *AGL23*, *AGL62*, *SHP1,2* and *STK*) (Liljegren et al., 2000; Pinyopich et al., 2003; Colombo et al., 2008; Kang et al., 2008; Hsu et al., 2014), and embryo and seed development (e.g. *DIA*, *AGL80*, *AGL23*, and *PHE1*) (Kohler et al., 2003; Portereiko et al., 2006; Bemer et al., 2008; Colombo et al., 2008). **(B)** Functions of MADS-box genes in orchid development. Orchid MADS-box proteins have been shown to regulate flowering and floral organ formation. *AG*, *AGAMOUS*; *AGL6*, *AGAMOUS-LIKE 6*; *AGL15*, *AGAMOUS-LIKE 15*; *AGL16*, *AGAMOUS-LIKE 16*; *AGL17*, *AGAMOUS-LIKE 17*; *AGL18*, *AGAMOUS-LIKE 18*; *AGL19*, *AGAMOUS-LIKE 19*; *AGL21*, *AGAMOUS-LIKE 21*; *AGL23*, *AGAMOUS-LIKE 23*; *AGL24*, *AGAMOUS-LIKE 24*; *AGL28*, *AGAMOUS-LIKE 28*; *AGL42*, *AGAMOUS-LIKE 42*; *AGL65*, *AGAMOUS-LIKE 65*; *AGL66*, *AGAMOUS-LIKE 66*; *AGL71*, *AGAMOUS-LIKE 71*; *AGL72*, *AGAMOUS-LIKE 72*; *AGL80*, *AGAMOUS-LIKE 80*; *AGL104*, *AGAMOUS-LIKE 104*; *ANR1*, *ARABIDOPSIS NITRATE REGULATED 1*; *AP1*, *APETALA1*; *AP3*, *APETALA3*; *CAL*, *CAULIFLOWER*; *CO*, *CONSTANS*; *DIA*, *DIANA*; *FLC*, *FLOWERING LOCUS C*; *FLM*, *FLOWERING LOCUS M*; *FT*, *FLOWERING LOCUS T*; *FUL, FRUITFULL*; *MAF2-5*, *MADS AFFECTING FLOWERING 2-5*; *PHE1*, *PHERES1*; *PI*, *PISTILLATA*; *SEP1-4, SEPALLATA1-4; SHP1,2*, *SHATTERPROOF1,2*; *SOC1*, *SUPPRESSOR OF OVEREXPRESSION OF CONSTANS 1*; *STK*, *SEEDSTICK*; *SVP*, *SHORT VEGETATIVE PHASE*; *XAL1*, *XAANTAL 1*; *XAL2*, *XAANTAL 2*.

The MIKC-type genes are specifically present in plants and many of these genes have been shown to control key processes of plant development including vegetative growth and reproductive organ development with complex cascades of events and networks (Theißen, 2001; Kaufmann et al., 2005; Adamczyk and Fernandez, 2009). Particularly, their functions in determining plant reproductive development are more remarkable as they regulate the development of consecutive reproductive processes, namely the floral transition, floral meristem specification, floral patterning, pollen growth, and development of ovules and seeds (**Figure 1**). The MADS-box genes that act in the regulation of flowering time and floral patterning in plants will be elaborated more in the latter sections (**Table 2**). The floral homeotic genes that play crucial roles in specifying reproductive floral organ identities are among the best characterized MADS-box genes. The extensive study of mutants with floral homeotic defects has resulted in the birth of the “ABCE model,” which explains how the genes of A, B, C, D and E classes act jointly to determine floral organs identities (Coen and Meyerowitz, 1991; Weigel and Meyerowitz, 1994; Theissen and Saedler, 2001). All floral homeotic genes in *Arabidopsis* belong to the MADS-box family except for the A-class gene, *APETALA2* (*AP2*). In addition to determining the identities of floral organs, MADS-box proteins also regulate floral meristem specification, the process of which involves four meristem identity genes, namely *LEAFY* (*LFY*) and three MADS-box genes closely related to each other, *APETALA1* (*AP1*), *CAULIFLOWER* (*CAL*), and *FRUITFULL* (*FUL*) (Ferrándiz et al., 2000; Ng and Yanofsky, 2001). Furthermore, studies of MADS-box proteins have also demonstrated their function in seed and silique growth. For example, three MADS-box genes, *SHATTERPROOF 1* (*SHP1*), and its close homologs *SHP2* and *SEEDSTICK* (*STK*) contribute to normal growth and development of carpels and fruits (Liljegren et al., 2000; Pinyopich et al., 2003). In addition, several MADS-box genes belonging to type I, such as *PHERES 1* (*PHE1*), *AGAMOUS-LIKE 80* (*AGL80*), *DIANA* (*AGL61*), etc., are involved in embryo and seed growth (Kohler et al., 2003; Portereiko et al., 2006; Bemer et al., 2008; Colombo et al., 2008; Kang et al., 2008).

**Table 2:** A summary of MADS-box regulators involved in reproductive development in the model plant *Arabidopsis* and orchids.

*Arabidopsis* gene name	Function	Orchid species	Orchid orthologs	References
*SUPPRESSOR OF OVEREXPRESSION OF CONSTANS* (*SOC1*)	Flowering promoter; FM^a^ specification; floral organ patterning	*Cymbidium goeringii*	*CgSOC1*	(Yang et al., 2019)
		*Dendrobium* Chao Praya Smile	*DOSOC1* ^b^	(Ding et al., 2013)
		*Dendrobium nobile*	*DnAGL19*	(Liang et al., 2012)
		*Orchis italica*	*Olcomp27839_SOC*	(Valoroso et al., 2019)
*SHORT VEGETATIVE PHASE* (*SVP*)	Flowering promoter; floral organ patterning	*C. goeringii*	*CgSVP1,* *CgSVP2* ^b^, *CgSVP3*	(Yang et al., 2019)
		*O. italica*	*Olcomp18466_SVP*	(Valoroso et al., 2019)
*FRUITFULL* (*FUL*)	Flowering promoter; FM specification; fruit development	*Dendrobium thyrsiflorum*	*DthyrFL1, DthyrFL2, DthyrFL3*	(Skipper et al., 2005)
		*Phalaenopsis hybrida* cv. Formosa rose	*ORAP11*, *ORAP13*	(Chen et al., 2007)
		*Phalaenopsis* hybrid “Athens”	*PhaMADS1, PhaMADS2*	(Acri-Nunes-Miranda and Mondragón-Palomino, 2014)
*APETALA 1* (*AP1*)	FM specification; sepal and petal identity	*Cymbidium ensifolium*	*ZHLZ.comp57026*	(Yang and Zhu, 2015)
		*Cymbidium faberi*	*CfAP11*	(Tian et al., 2013)
		*C. goeringii*	*CgAP1*	(Yang et al., 2019)
		*Dendrobium* Chao Praya Smile	*DOAP1* ^b^	(Sawettalake et al., 2017)
		*Dendrobium* Madame Suzie Wong	*DOMADS2*	(Yu and Goh, 2000)
		*Oncidium* Gower Ramsey	*OMADS10 (OAP1)*	(Chang et al., 2009; Hsu et al., 2015)
		*O. italica*	*Olcomp2508_AP1, Olcomp3679_AP1,* *Olcomp9283_AP1, Olcomp11046_AP1*	(Valoroso et al., 2019)
		*Phalaenopsis aphrodite*	*PaAP1-1, PaAP1-2*	(Su et al., 2013b)
*APETALA3* (*AP3*)	Petal and stamen identity	*C. ensifolium*	*CeAP3, ZHLH.comp53790, ZHLZ.comp35346, ZHLZ.comp55590, ZHLZ.comp26961*	(Yang and Zhu, 2015)
		*Cymbidium* hybrid cultivar	*MADS1*	(Aceto and Gaudio, 2011)
		*Dendrobium crumenatum*	*DcOAP3A; DcOAP3B*	(Xu et al., 2006)
		*Dendrobium moniliforme*	*DMMADS4*	(Aceto and Gaudio, 2011)
		*Gongora galeata*	*GogalDEF1, GogalDEF2, GogalDEF3*	(Aceto and Gaudio, 2011)
		*Habenaria radiata*	*HrDEF*	(Aceto and Gaudio, 2011)
		*Oncidium* Gower Ramsey	*OMADS3* (*OAP3-3*)	(Hsu and Yang, 2002; Hsu et al., 2015)
			*OMADS5* (*OAP3-1*), *OMADS9* (*OAP3-2*)	(Chang et al., 2010; Hsu et al., 2015)
			*OMADS12* (*OAP3-4*)	(Hsu et al., 2015)
		*O. italica*	*Olcomp900_DEF4, Olcomp3831_DEF1,* *Olcomp7668_DEF3, Olcomp22604_DEF2*	(Valoroso et al., 2019)
		*P. aphrodite*	*PaAP3-1, PaAP3-2, PaAP3-3, PaAP3-4*	(Su et al., 2013b)
		*Phalaenopsis equestris*	*PeMADS2, PeMADS3 PeMADS4*	(Tsai et al., 2004)
			*PeMADS5* ^b^	(Tsai et al., 2004; Hsieh et al., 2013a)
		*Phragmipedium longifolium*	*PhlonDEF1, PhlonDEF2, PhlonDEF3, PhlonDEF4*	(Aceto and Gaudio, 2011)
		*Spiranthes odorata*	*SpodoDEF1, SpodoDEF2, SpodoDEF3*	(Aceto and Gaudio, 2011)
		*Vanilla planifolia*	*VaplaDEF1, VaplaDEF2, VaplaDEF3*	(Aceto and Gaudio, 2011)
*PISTILLATA* (*PI*)	Petal and stamen identity	*D. crumenatum*	*DcOPI*	(Xu et al., 2006)
		*Dendrobium thyrisiflorum*	*DthyrPI*	(Aceto and Gaudio, 2011)
		*Epipactis palustris*	*EpalPI*	(Aceto and Gaudio, 2011)
		*G. galeata*	*GogalGLO1*	(Aceto and Gaudio, 2011)
		*H. radiata*	*HrGLO1, HrGLO2*	(Aceto and Gaudio, 2011)
		*Oncidium* Gower Ramsey	*OMADS8 (OPI)*	(Chang et al., 2009; Hsu et al., 2015; Mao et al., 2015)
		*O. italica*	*Olcomp1173_PI, Olcomp1989_PI*	(Aceto and Gaudio, 2011; Valoroso et al., 2019)
		*P. aphrodite*	*PaPI-1*	(Su et al., 2013b)
		*P. equestris*	*PeMADS6* ^b^	(Hsieh et al., 2013a, Hsieh et al., 2013b, Tsai et al., 2005; Lu et al., 2007)
		*Phragmipedium longiflorum*	*PhlonGLO1*	(Aceto and Gaudio, 2011)
		*S. odorata*	*SpodoGLO1*	(Aceto and Gaudio, 2011)
		*V. planifolia*	*VaplaGLO1*	(Aceto and Gaudio, 2011)
*AGAMOUS* (*AG*)	Stamen and carpel identity; floral meristem determinacy	*C. ensifolium*	*CeMADS1, CeMADS2,* *ZHLZ.comp46850,* *ZHLZ.comp52597,* *ZHLZ.comp58360, ZHLZ.comp52003,* *ZHLZ.comp50822*	(Wang et al., 2011; Yang and Zhu, 2015)
		*D. crumenatum*	*DcOAG1*	(Xu et al., 2006)
		*D. thyrsiflorum*	*DthyrAG1*	(Skipper et al., 2006)
		*Oncidium* Gower Ramsey	*OMADS4*	(Hsu et al., 2010)
		*O. italica*	*Olcomp1784_AG, Olcomp7958_AG, Olcomp16674_AG*	(Valoroso et al., 2019)
		*P. aphrodite*	*PaAG-1, PaAG-2, PaAG-3*	(Su et al., 2013b)
		*P. equestris*	*PeMADS1*	(Chen et al., 2012; Hsieh et al., 2013b)
		*Phalaenopsis* hybrid “Athens”	*PhaMADS8, PhaMADS10*	(Acri-Nunes-Miranda and Mondragón-Palomino, 2014)
		*Phalaenopsis* sp. “Hatsuyuki”	*PhalAG1*	(Song et al., 2006)
*SEEDSTICK* (*STK*)	Ovule and seed integument identity	*D. crumenatum*	*DcOAG2*	(Xu et al., 2006)
		*D. thyrsiflorum*	*DthyrAG2*	(Skipper et al., 2006)
		*Oncidium* Gower Ramsey	*OMADS2*	(Hsu et al., 2010)
		*O. italica*	*Olcomp3859_STK*	(Valoroso et al., 2019)
		*P. aphrodite*	*PaAG-4*	(Su et al., 2013b)
		*P. equestris*	*PeMADS7*	(Chen et al., 2012; Hsieh et al., 2013b)
		*Phalaenopsis* hybrid “Athens”	*PhaMADS10*	(Acri-Nunes-Miranda and Mondragón-Palomino, 2014)
		*Phalaenopsis* sp. “Hatsuyuki”	*PhalAG2*	(Song et al., 2006)
*SEPALLATA*s (*SEP*s)	Floral organ identity; flowering time regulation	*C. ensifolium*	*CeSEP3, ZHLZ.comp51896, ZHLZ.comp57688, ZHLZ.comp57446, ZHLZ.comp58442*	(Yang and Zhu, 2015)
		*D. crumenatum*	*DcOSEP1*	(Xu et al., 2006)
		*Dendrobium* Madame Suzie Wong	*DOMADS1, DOMADS3*	(Yu and Goh, 2000)
		*Oncidium* Gower Ramsey	*OMADS6 (OSEP3) OMADS11 (OSEP1)*	(Chang et al., 2009; Hsu et al., 2015)
		*O. italica*	*Olcomp1006_SEP, Olcomp7010_SEP*	(Valoroso et al., 2019)
		*P. aphrodite*	*PaSEP-1*, *PaSEP-2*, *PaSEP-3*	(Su et al., 2013b)
		*P. equestris*	*PeSEP1, PeSEP2* ^b^ *, PeSEP3* ^b^ *, PeSEP4*	(Pan et al., 2014)
		*Phalaenopsis* hybrid “Athens”	*PhaMADS4, PhaMADS5, PhaMADS7*	(Acri-Nunes-Miranda and Mondragón-Palomino, 2014)
*AGAMOUS-LIKE 6* (*AGL6*)	Flowering promoter	*Oncidium* Gower Ramsey	*OMADS7 (OAGL6-1)* *OMADS1* *^b^* * (OAGL6-2)*	(Chang et al., 2009; Hsu et al., 2015)
		*O. italica*	*Olcomp1386_AGL6, Olcomp4335_AGL6, Olcomp8204_AGL6*	(Valoroso et al., 2019)
		*P. aphrodite*	*PaAGL6-1*, *PaAGL6-2*	(Su et al., 2013b)

a FM, floral meristem.

b MADS-box genes whose function has been examined by stable or transient overexpression or silencing in orchids.

## Mads-Box Proteins in Orchid Flowering

### The Floral Transition of Orchid

The floral transition, a developmental transition from vegetative to reproductive phase, is one of the key developmental transitions in the plant life cycle. The timing of floral transition greatly affects the success of plant reproduction. In the model plant *Arabidopsis*, the vegetative shoot apex, from which leaves are generated, is converted into the inflorescence meristem, from which flowers are generated, during the floral transition. In orchids, the length of the vegetative phase can vary from one to thirteen years between different species, but the average time for most species is between two to three years (Hew and Yong, 2004). The process of flowering is induced in the meristem of dormant axillary buds. In sympodial orchids such as *Dendrobium* and *Oncidium*, the formation of bud primordia occurs at the axils of the leaves. In the orchid *Cymbidium*, the inflorescence will be developed from the dormant axillary buds at the base of the pseudobulb. For monopodial orchids such as *Phalaenopsis* and *Vandas*, they typically have at least two dormant bud primordia at each leaf axil which can grow into inflorescences or keikis (new orchid plantlets).

With the increasing demand for whole orchids and cut flowers, modern horticulturists and breeders are learning about the flowering behavior of different species to control the time of blooming so as to maximize their economic value. Each orchid species has a time of the year when it will bloom naturally. Most orchids will grow in the abundance of sunlight and moisture during summer and bloom in the fall, winter, or spring. Significant progress has been made to determine the effects of environmental conditions, such as temperature and day length, in inducing flowering in different species (**Table 3**). Studies on *Cymbidium*, *Oncidium*, *Dendrobium*, and *Phalaenopsis* have shown that a low night temperature of 13°C and a large fluctuation of 10°C to 14°C in daily diurnal temperature are sufficient to induce flowering (Rotor, 1952; Rotor and Withner, 1959). A high day temperature of more than 28°C for 8 h or longer promotes vegetative growth and inhibit the process of flowering in *Phalaenopsis* (Blanchard and Runkle, 2006; Newton and Runkle, 2009). However, a prolonged exposure to a constant elevated temperature of 30°C induces flowering by activating the thermal stress response (Chin et al., 2014). Since orchids are likely to be shaded by leaves on trees, the length of daylight is not known to influence flowering with the exception of *Dendrobium phalaenopsis* flowering under short days (Rotor, 1952; Rotor and Withner, 1959; Lopez and Runkle, 2004). It is noteworthy to mention that flowering is an intricate process and many environmental conditions including light intensity and humidity can affect the initiation of floral spikes.

**Table 3:** The promotive environmental factors for orchid flowering.

Genus	Temperature	Photoperiod	References
*Cymbidium*	Low night temperature of 13°C A difference of 10°C–14°C in diurnal temperature	No known influence	(Rotor, 1952; Rotor and Withner, 1959; Goh et al., 1982; Powell et al., 1988; An et al., 2012)
*Oncidium*	Low night temperature A large difference in diurnal temperature High constant temperature (30°C) for 2 weeks	No known influence	(Chang and Lee, 2000; Chin et al., 2014)
*Dendrobium*	Low night temperature of 13°C 3 weeks at 13°C to 15°C	Flowering under short days (for *Dendrobium Phalaenopsis* only)	(Rotor 1952; Rotor and Withner 1959; Goh et al., 1982; Sinoda et al., 1988; Lopez and Runkle, 2004)
*Phalaenopsis*	Day temperature not higher than 28°C Night temperature of 15°C to 18°C	No known influence	(Tran Thanh Van, 1974; Nishimura et al., 1976; Sakanishi et al., 1980; Baker and Baker, 1996; Blanchard and Runkle, 2006; Newton and Runkle, 2009)

### Floral Pathway Integrators

**Figure 2:**
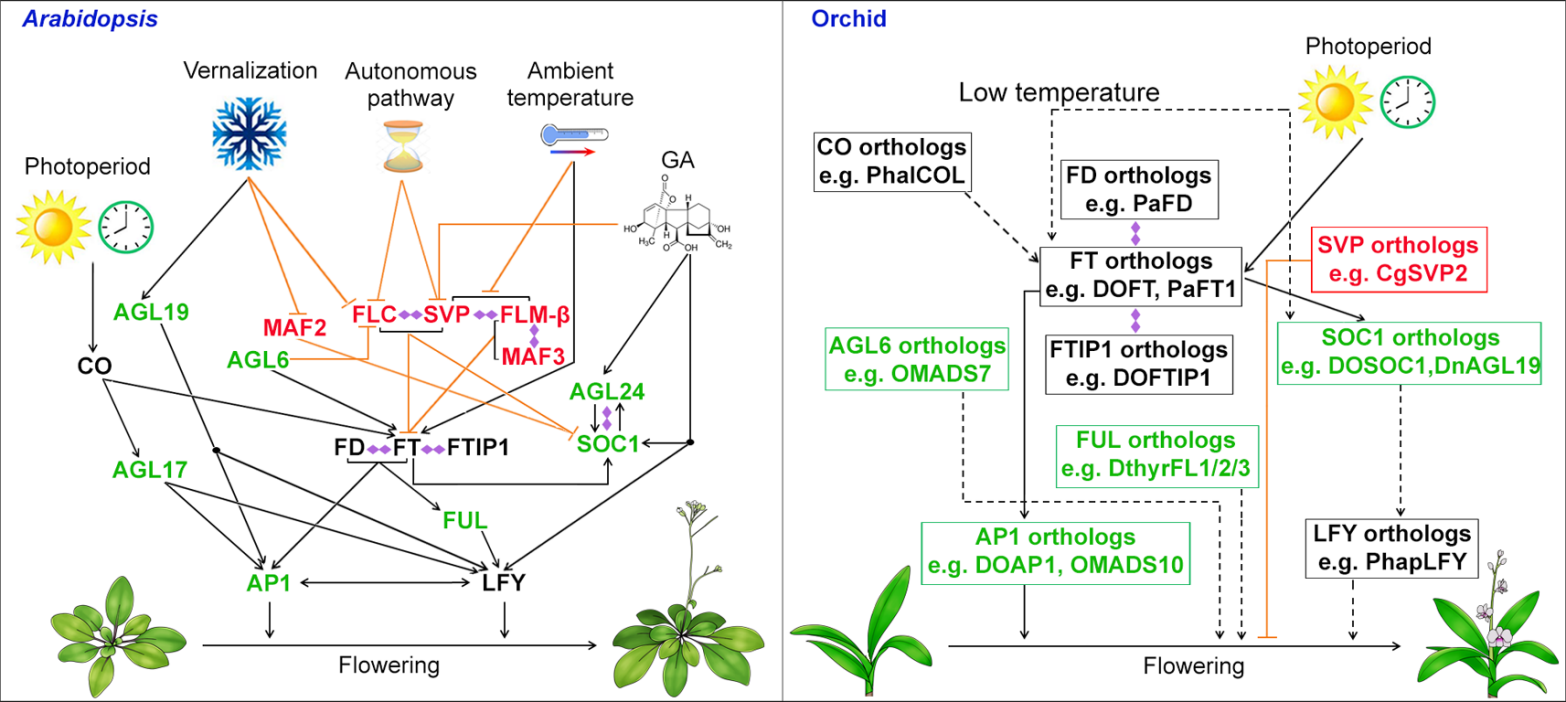
Biological roles of MADS-box genes in controlling flowering in the model plant *Arabidopsis* and orchid. In *Arabidopsis*, the MADS-box genes including *SOC1*, *FLC*, *SVP* and *AGL24* integrates signals for flowering from environmental and endogenous cues. In orchid, orthologous genes of *SOC1*, *AGL6*, *SVP*, and *AP1* have been isolated and functionally characterized either in heterologous system (e.g. *Arabidopsis*) or orchid and shown to be involved in promoting flowering. MADS-box transcription factors that function as flowering activators and suppressors are shown in green and red, respectively, whereas other flowering regulators are shown in black. Promoting and repressive effects are indicated by black arrows and orange T bars, respectively. The dashed lines with arrows indicate possible positive regulation based on the studies using heterologous systems. Double-ended diamond arrows indicate protein–protein interactions. AGL6, AGAMOUS-LIKE 6; AGL17, AGAMOUS-LIKE 17; AGL19, AGAMOUS-LIKE 19; AGL24, AGAMOUS-LIKE 24; AP1, APETALA1; CO, CONSTANS; FLC, FLOWERING LOCUS C; FLM, FLOWERING LOCUS M; FT, FLOWERING LOCUS T; FTIP1, FT-INTERACTING PROTEIN 1; FUL, FRUITFULL; LFY, LEAFY; MAF2, MADS AFFECTING FLOWERING 2; SOC1, SUPPRESSOR OF OVEREXPRESSION OF CONSTANS 1; SVP, SHORT VEGETATIVE PHASE.

For the model plant *Arabidopsis*, the timing of floral transition is regulated by a complex system consisting of several flowering pathways—photoperiod, vernalization, thermosensory, gibberellins (GA), autonomous, and age—that perceive both environmental and endogenous flowering signals (Mouradov et al., 2002; Simpson and Dean, 2002; Blázquez et al., 2003; Boss et al., 2004; Wang et al., 2009). For environmental flowering signals, the photoperiod pathway perceives the daylength in seasonal changes; the vernalization pathway measures the period of the plant’s exposure to cold; the thermosensory pathway mediates the ambient temperature effect. For endogenous flowering signals, the GA pathway promotes flowering under non-inductive photoperiod, while the autonomous pathway is a photoperiod-independent pathway that induces flowering through perceiving the internal signals at various stages of development. These genetic pathways regulate the transcription of two main integrators of floral pathways, *FLOWERING LOCUS T* (*FT*) and the MADS-box gene *SUPPRESSOR OF OVEREXPRESSION OF CONSTANS 1* (*SOC1*), which then activate the expression of *AP1* and *LFY*, two floral meristem identity genes, to start the process of floral meristem formation (**Figure 2**) (Kardailsky et al., 1999; Kobayashi et al., 1999; Blázquez and Weigel, 2000; Lee et al., 2000; Samach et al., 2000; Liu et al., 2009). Several MADS-box proteins including SOC1, AGAMOUS-LIKE 24 (AGL24), AGL6, and AGL17, promote flowering (Lee et al., 2000; Yu et al., 2002; Han et al., 2008; Yoo et al., 2011), whereas MADS-box regulators including FLOWERING LOCUS C (FLC), SHORT VEGETATIVE PHASE (SVP), MADS AFFECTING FLOWERING 1/FLOWERING LOCUS M (MAF1/FLM), and MAF2/3/4/5 form various complexes to repress flowering (**Figure 2**) (Helliwell et al., 2006; Searle et al., 2006; Li et al., 2008; Gu et al., 2013; Lee et al., 2013; Pose et al., 2013; Mateos et al., 2015). Another three MADS-box transcription factors that are closely related to each other, AP1, CAL, and FUL are also involved in the activation of *LFY* in promoting flowering and floral meristem specification as the triple mutant of these genes generates leafy shoots in place of floral organs (Ferrándiz et al., 2000).

In orchids, the process of the floral transition occurs in the axillary buds where the bud primordia will develop into a more convex shape upon entering reproductive phase. Recent works have identified and examined many orthologs of flowering integrators and other MADS-box genes in orchids (**Figure 2**). As one of the major floral pathway integrator genes, *FT*, encoding a small globular protein, is transcriptionally activated by CONSTANS (CO) in companion cells in the leaf veins and the FT protein moves to the shoot apical meristem (Kardailsky et al., 1999; Kobayashi et al., 1999; An et al., 2004; Corbesier et al., 2007; Liu et al., 2012; Nakamura et al., 2014; Zhu et al., 2016). In orchids for instance, *Oncidium*, *Dendrobium*, and *Cymbidium*, the expression of *FT* orthologs was predominantly expressed in the leaves and axillary buds. In addition, the expression of *FT* orthologs has been found to be influenced by daylength in *Oncidium* and *Cymbidium*, showing a similar photoperiodic pattern like *FT* in *Arabidopsis* (Hou and Yang 2009; Huang et al., 2012). Ectopic expression of *FT* orthologs, *OnFT*, *DnFT*, *DOFT*, *CeFT*, *CgFT*, *CsFT*, and *PaFT1* from the orchids *Oncidium*, *Dendrobium*, *Cymbidium*, and *Phalaenopsis*, respectively, results in a precocious flowering phenotype in transgenic plants of *Arabidopsis* or tobacco (Hou and Yang, 2009; Huang et al., 2012; Li et al., 2012; Xiang et al., 2012; Jang, 2015; Wang et al., 2017). More importantly, downregulation of *DOFT* delays flowering in *Dendrobium* orchids, whereas overexpression of *DOFT* accelerates flowering in orchids (Wang et al., 2017). Interestingly, low temperature treatment specifically induces the expression of *FT* in leaves in both *Dendrobium* and *Phalaenopsis*, suggesting *FT* is the main floral inducer under floral inductive low temperature regime (Li et al., 2012; Jang et al., 2015).

### MADS-Box Genes and Orchid Flowering

*SOC1* encodes a MADS-box transcription factor that is a member of the *Tomato MADS-box gene 3* (*TM3*)-like genes subfamily from angiosperms and gymnosperms (Lee et al., 2000; Becker and Theißen, 2003; Cseke et al., 2003; Nakamura et al., 2005). *SOC1* expression is detected in both leaves and shoot apices and is regulated by several floral pathways (Borner et al., 2000; Lee et al., 2000; Samach et al., 2000; Moon et al., 2003). In *Dendrobium nobile*, the expression of a close *SOC1* ortholog, *DnAGL19*, has been found to be increased after vernalization (Liang et al., 2012). In the orchid *Dendrobium* Chao Praya Smile, the expression of the *SOC1* ortholog *DOSOC1* is highly detected in reproductive organs, such as inflorescence apex, pedicel, floral buds and open flowers (Ding et al., 2013). *DOSOC1* expression is upregulated in the whole seedlings upon the floral transition (Ding et al., 2013). Overexpression of *DOSOC1* shows early flowering in both *Arabidopsis* and *Dendrobium* orchids, implying the evolutionary conserved functions of *SOC1*-like genes as activators of flowering (**Figure 2**) (Ding et al., 2013). Moreover, *DOSOC1* expression is downregulated in *DOFT* knockdown *Dendrobium* orchid, whereas its expression is upregulated in *DOFT* overexpression orchid (Wang et al., 2017), indicating a conserved regulatory mechanism of *SOC1*-like genes expression. Intriguingly, *DOSOC1* overexpression in *Dendrobium* results in abnormal floral organ development with formation of immature perianth organs only, indicating the role of *DOSOC1* in maintaining the identity of floral meristem and formation floral organs (Ding et al., 2013). This is in line with the function of *SOC1*-like genes in flower development in some plant species (Liu et al., 2013; Teo et al., 2014). Additionally, another MADS-box gene *FUL* has been shown to act redundantly with *SOC1* in regulation of flowering time in *Arabidopsis* (Melzer et al., 2008). Mutations in *ful* only slightly delay flowering, while in combination with *soc1* mutants, the flowering is further delayed as compared with both single mutants. Three *FUL*-like genes have been isolated in the orchid *Dendrobium thyrsiflorum*, namely *DthyrFL1/2/3* (Skipper et al., 2005). These three genes are upregulated during orchid reproductive development, yet their involvement in orchid flowering remains unknown.

*SOC1* expression is repressed by a floral repressor protein complex formed by FLC and SVP, which are also MADS-box proteins (Li et al., 2008). The orchid *SVP* orthologs have been reported in *Cymbidium* orchids, whereas no *FLC* orthologs have been isolated so far in monocots. The expression levels of *CgSVP1/2/3*, *SVP* orthologs, are greatly reduced upon cold treatment in *Cymbidium goeringii* (Yang et al., 2019). Moreover, transient overexpression of *CgSVP2* results in retarded flower bud growth, indicating its role as a repressor of flower bud formation (Yang et al., 2019). However, the involvement of *SVP* orthologs in orchid floral transition and its potential regulation of *SOC1* need further investigation.

*AGL6* encodes another MADS-box transcription factor which regulates the transition to flowering in *Arabidopsis* (Yoo et al., 2011). Knockdown of *AGL6* by artificial microRNA leads to late flowering, in contrast, *agl6-1D* wherein *AGL6* is activated by the *35S* enhancer shows early flowering. Two *AGL6*-like genes, *OMADS1* (*OAGL6-2*) and *OMADS7* (*OAGL6-1*), have been found in the *Oncidium* Gower Ramsey orchid, and overexpression of either gene leads to early flowering in *Arabidopsis* (Hsu et al., 2003; Chang et al., 2009), implying a conserved function of *AGL6*-like genes in mediating flowering.

As mentioned above, AP1, a MADS-box protein, specifies the identity of floral meristem as well as sepal and petal identity (Irish and Sussex, 1990; Mandel et al., 1992; Bowman et al., 1993). In *ap1* mutants, flowers exhibit a homeotic transformation that sepals develop into bracts and petals fail to develop. By comparison, *AP1* overexpression causes early flowering and conversion of the inflorescence meristem into a determinate floral meristem (Mandel and Yanofsky, 1995). Orthologs of *AP1* have been identified and characterized in *Oncidium*, *Dendrobium*, and *Cymbidium*. The expression of *AP1* orthologs can be detected in both vegetative tissues and reproductive structures such as floral buds and pedicel (Yu and Goh, 2000; Chen et al., 2007; Chang et al., 2009; Tian et al., 2013; Sawettalake et al., 2017). Overexpression of *AP1* orthologs, such as *OMADS10* from *Oncidium* Gower Ramsey and *DOAP1* from *Dendrobium* Chao Praya Smile, causes early flowering as well as conversion of inflorescence meristems to determinate floral meristems in *Arabidopsis* (Chang et al., 2009; Sawettalake et al., 2017). Transgenic *Dendrobium* orchids overexpressing *DOAP1* also show accelerated flowering as compared to wild-type orchid and conversion of inflorescence meristems to determinate floral meristems (Sawettalake et al., 2017). In addition, *DOAP1* expression is promoted by *DOFT* in *Dendrobium* orchids, which is a conserved regulation as that in *Arabidopsis* (Wang et al., 2017). These studies suggest that orchid *AP1* orthologs have conserved functions in promoting the floral transition and determination of floral meristems.

## Mads-Box Proteins in Orchid Floral Patterning

### The Orchid Flower

**Figure 3:**
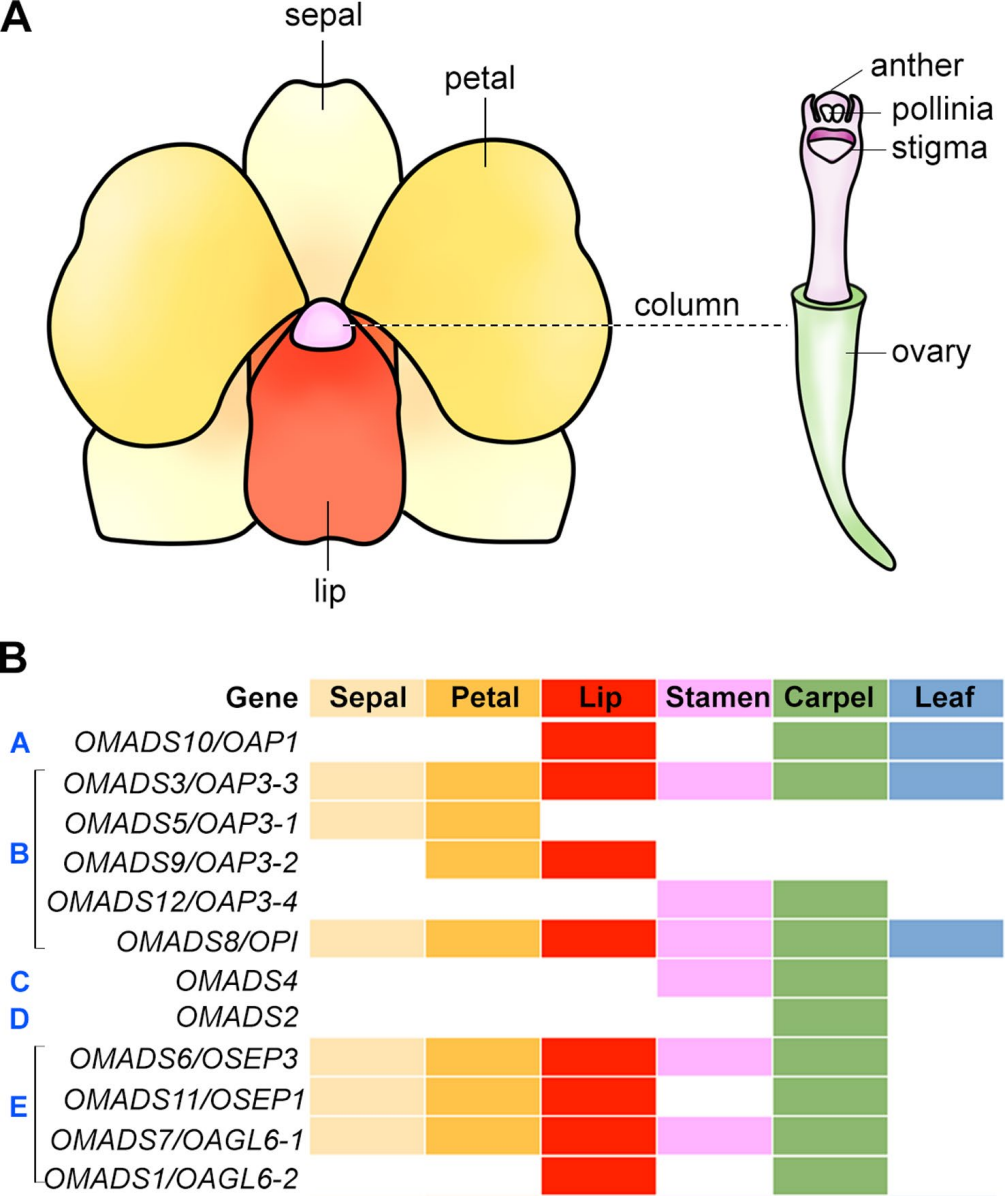
Floral organ identity genes in orchid. **(A)** An illustration showing a typical orchid flower structure. **(B)** Expression patterns of orthologs of floral organ identity genes in orchid. The floral organs, sepal, petal, lip, stamen, carpel, and leaf are color-coded, and presence of these colors indicates detected expression in these organs. The white color indicates no expression detected. The gene expression patterns are shown based on the studies in the *Oncidium* orchid (Chang et al., 2009; Chang et al., 2010; Hsu et al., 2010; Hsu et al., 2015).

In angiosperms, the flowers are usually composed of four types of structures, which form two parts, namely the vegetative part and the reproductive part. While the morphology and elaboration can differ greatly among different species, the diversification of floral patterning has taken place in a relatively conserved manner in having similar general organization of four types of structures arranged in four concentric whorls. In *Arabidopsis*, the flower consists of four concentric whorls of floral organs from the outer to inner whorls: sepals (four), petals (four), stamens (six), and two fused carpels. In orchids, the flowers are usually bilaterally symmetrical (zygomorphic) with three outer sepals, two inner petals, and a highly specialized inner median petal named lip or labellum which acts as the main pollinator attractant (**Figure 3A**). In a number of orchid species, the outer sepals and inner petals are named as tepals as they cannot be distinguished from each other morphologically. The reproductive structure gynostemium or column is composed of fused male (stamen/anther) and female (carpel/pistil) organs.

### ABCE and Floral Quartet Model

**Figure 4:**
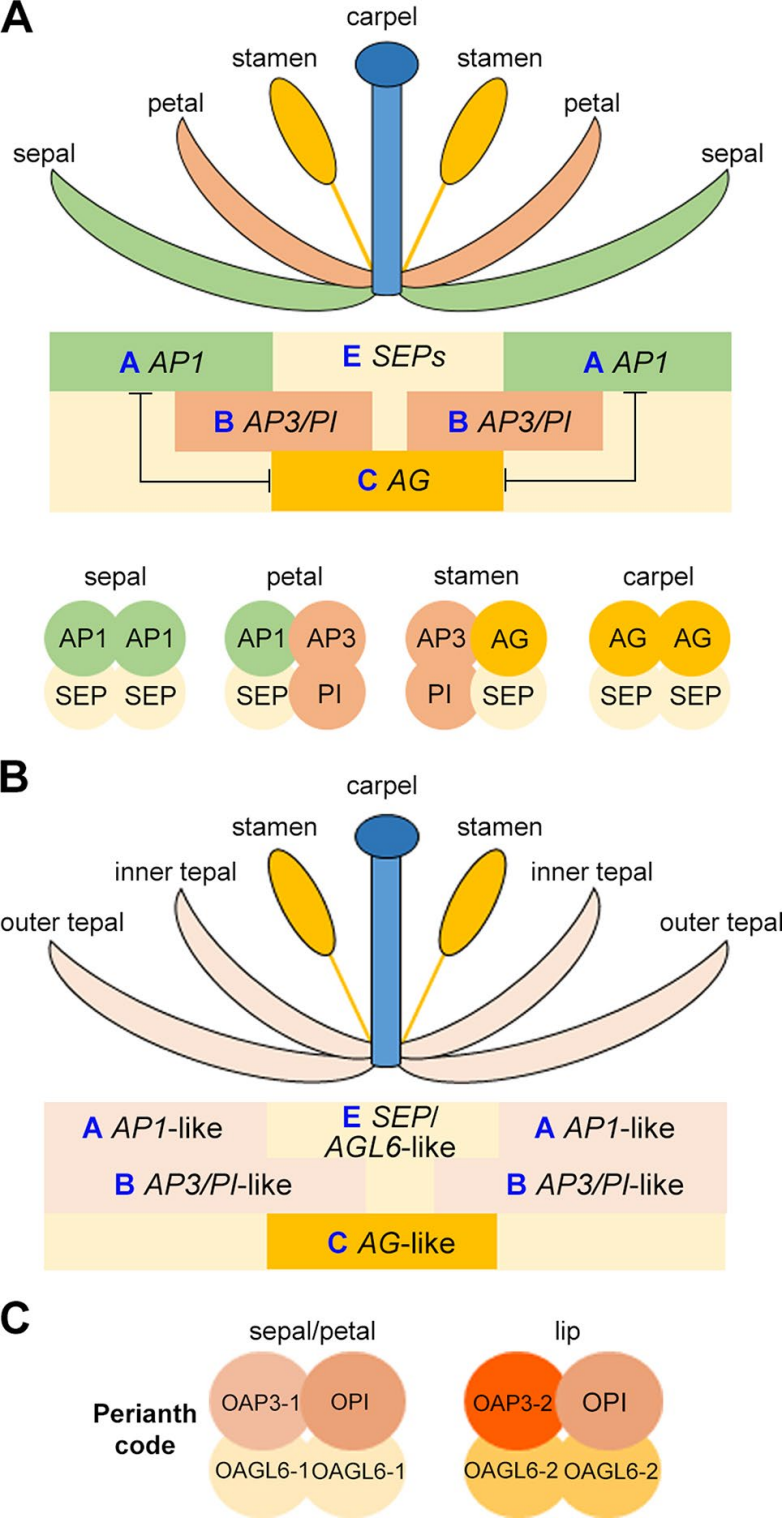
Schematic drawing showing the ABCE model **(A)**, the modified ABC model **(B)**, and the orchid Perianth code **(C)**.

Understanding the specification of the distinct floral organs through genetic study in *Arabidopsis* and *Antirrhinum majus* (snapdragon) has resulted in the birth of “ABCE model” and “floral quartet model” (**Figure 4A**) (Meyerowitz et al., 1989; Schwarz-Sommer et al., 1990; Coen and Meyerowitz, 1991; Weigel and Meyerowitz, 1994; Irish, 2010; Theißen et al., 2016). In the classical “ABC model,” a combination of three gene classes specifies the four types of floral organs: sepal, petal, stamen, and carpel (Coen and Meyerowitz, 1991; Weigel and Meyerowitz, 1994). In *Arabidopsis*, the A-class genes (*AP1* and *AP2*) determine the sepal identify in the outermost whorl, A-class and B-class (*AP3* and *PI*) genes together specify petals in the second whorl, B-class and C-class (*AG*) genes determine the identity of the male reproductive organ stamen in the third whorl, and the C-class gene specifies the female reproductive organ carpel in the innermost whorl. The expression domains of A-class and C-class genes are mutually exclusive. After discovering that the E-class genes (*SEP1–4*) are essential for the determination of all of the four whorls of floral organs, the classical “ABC model” was then extended to the “ABCE model.” Additionally, D-class genes (*STK* and *SHP1/2*) are needed for determining the identity of ovule (Pinyopich et al., 2003). Interestingly, except for *AP2,* all these genes belong to MADS-box gene family, and their proteins form tetrameric complexes. This was coined as the “floral quartet model” as the tetrameric complexes functions as a whole to direct the development of specific floral organs (**Figure 4A**) (Theissen and Saedler, 2001). In orchids, the study of MADS-box transcription factors related to floral patterning has been challenging as recent functional analyses have shown that these genes have functionally diversified in their own lineages, making the prediction of function on the basis of orthology difficult (Irish and Litt, 2005).

### MADS-Box Genes and Orchid Floral Patterning

#### A-Class Genes

In orchids, there are several *AP1* orthologs isolated in *Cymbidium*, *Oncidium*, *Dendrobium*, and *Phalaenopsis* (**Table 2**). In *Dendrobium Madame Thong-In*, the *AP1* ortholog *DOMADS2* is expressed during the transitional phase and floral development (Yu and Goh, 2000). In mature flowers, *DOMADS2* is detected in the column and ovary but not in the pedicel, sepal, or petal. The differential expression pattern compared with *AP1* expression in the sepal and petal in *Arabidopsis* suggests the functional divergence of *AP1*-like genes during floral patterning. In the *Dendrobium* Chao Praya Smile orchid, the *AP1* ortholog *DOAP1* is detected at high levels in the inflorescence meristem as well as flowers. In addition, the overexpression of *DOAP1* can partially complement the *Arabidopsis ap1* mutant in restoring petal formation, suggesting *DOAP1* functions as a homeotic gene (Sawettalake et al., 2017). In *Oncidium* Gower Ramsey, the *AP1* orthologs *OMADS10* is expressed in the leaves, lip and carpel (**Figure 3B**). Overexpression of *OMADS10* induces early flowering without any floral organs defects in *Arabidopsis* (Chang et al., 2009). Two *AP1* orthologs, *PaAP1-1* and *PaAP1-2* has been isolated in the moth orchid *Phalaenopsis aphrodite* (Su et al., 2013b). *PaAP1-1* is mainly expressed in the pollinia and pedicel, whereas *PaAP1-2* is specifically expressed in the pedicel. The expression patterns of orchid *AP1* orthologs are unlike the A-class genes in *Arabidopsis* which are only present in the sepals and petals, but is somehow similar to the monocot lily *AP1* orthologs, *LMADS5/6*, which are expressed in the vegetative leaves and the innermost whorl carpel (Chen et al., 2008). This indicates the divergent function of orchid *AP1*-like genes in floral organ development.

#### B-Class Genes

The B-class genes are necessary for determining the identity of petals and stamens. There are two B-class genes in *Arabidopsis*, *AP3* and *PI,* analogous to the *A. majus DEFICIENS* and *GLOBOSA*, respectively. Mutations in either *AP3* or *PI* lead to similar phenotypes wherein petals and stamens are transformed into sepals and carpels, respectively (Bowman et al., 1989; Hill and Lord, 1989). Many studies have identified various numbers of B-class genes and studied their expression patterns in several orchid species (**Table 2**) (Hsu and Yang, 2002; Tsai et al., 2004; Tsai et al., 2005; Xu et al., 2006; Mondragón-Palomino and Theißen, 2008; Chang et al., 2009; Chang et al., 2010; Su et al., 2013b; Tsai et al., 2014; Hsu et al., 2015; Mao et al., 2015; Yang and Zhu, 2015). In *Dendrobium crumenatum*, the expression of *DcOAP3A* and *DcOPI* is detected in all parts of the mature flowers, but the expression of *DcOAP3B* is present in petals, lips, anthers, and column only (Xu et al., 2006). DcOAP3A/B can form heterodimers with DcOPI. In *Oncidium* Gower Ramsey, *OMADS3* (*OAP3-3*), *OMADS5* (*OAP3-1*), *OMADS9* (*OAP3-2*), and *OMADS12* (*OAP3-4*) belong to the *AP3* lineage, while *OMADS8* (*OPI*) belongs to the *PI* lineage. *OMADS3* and *OMADS8* are expressed in both vegetative tissues and all floral organs of mature flowers (**Figure 3B**) (Chang et al., 2010). *OMADS5* is detected in both sepals and petals but not in the lip, whereas *OMADS9* is detected in the petals and lips (Chang et al., 2010). *OMADS12* is detected in the orchid reproductive floral organs including stamens and carpels, but not in the sepals, petals, and lips (Hsu et al., 2015). Overexpression of truncated *OMADS3* in *Arabidopsis* results in *ap2*-like flowers with homeotic conversion from sepals and petals to carpel-like and stamen-like organs (Hsu and Yang, 2002), while overexpression of *OMADS8,* but not *OMADS5/9,* causes the transformation of sepals to expanded petal-like structures (Chang et al., 2010). While OMADS3, OMADS5, and OMADS9 can assemble into both homodimers and heterodimers within the same group, OMADS8 can only form heterodimer with OMADS3 (Chang et al., 2010). It has been proposed that *OMADS3/5/8/9* is probably needed for the specification of sepals and petals and *OMADS3/8/9* but the absence of *OMADS5* leads to the formation of lips (Chang et al., 2010). In *P. equestris*, the MADS-box genes *PeMADS2*, *PeMADS3*, *PeMADS4,* and *PeMADS5* belong to the *AP3* lineage and *PeMADS6* belongs to the *PI* lineage. They are all expressed in lips and columns with *PeMADS2* also found in sepals and petals and *PeMADS3* in petals (Tsai et al., 2004; Tsai et al., 2005). Similarly to those B-class genes in *Dendrobium* and *Oncidium*, PeMADS2-5 interacts with PeMADS6 to form heterodimers and binds to CarG boxes on DNA (Tsai et al., 2005).

#### C- and D-Class Genes

In *Arabidopsis*, the C-class gene *AG* is required for the normal development of the stamens and carpels found in the third and fourth whorls, respectively. Mutations in *AG* cause the homeotic transformation of stamens and carpels into petals and sepals (Yanofsky et al., 1990). In addition, since *AG* is also necessary for floral meristem determinacy, the flowers of *ag* mutants are indeterminate and show the “flower within a flower” phenotype of sepal-petal-petal reiteration. D-class genes are required for regulating ovule identify. C and D-class genes are members of the *AG*-like family and are resulted from an ancient gene duplication event (Becker and Theißen, 2003). Both C and D-class genes have been identfied from several orchid speices (**Table 2**).

In *P. equestris*, *Phalaenopsis* sp. “Hatsuyuki,” *Cymbidium ensifolium*, and *Oncidium* Gower Ramsey, the C-class genes *PeMADS1*, *PhalAG1*, *CeMADS1*, and *OMADS4,* respectively, are highly expressed in the floral buds and column in mature flowers (**Figure 3B**) (Song et al., 2006; Hsu et al., 2010; Wang et al., 2011; Chen et al., 2012). *OMADS4* in the *Oncidium* Gower Ramsey orchid is specifically detected in the stamens and carpels, similar to the expression pattern of LMADS10 from Lilium longiflorum (Hsu et al., 2010). Both *OMADS4* and *LMADS10*, when overexpressed in *Arabidopsis*, result in early flowering, whereas *LMADS10* overexpression also leads to curly leaves and floral organ conversions, indicating the probable functional diversification of the monocot C-class genes. Several C-class genes from other orchid species have broader expression pattern. For examples, the *D. crumenatum* and *D. thyrsiflorum AG* orthologs, *DcOAG1* and *DthyrAG1*, respectively, are expressed in all kinds of floral organs and are not confined to the reproductive organs (Skipper et al., 2006; Xu et al., 2006). This expression pattern is similar to the *AG* homolog from *Illicium floridanum* that is also expressed in the tepals and reproductive organs (Kim et al., 2005), suggesting the regulatory mechanisms involved in the regulation of the expression of these C-class genes have evolved independently. The ectopic expression of *DcOAG1* in *Arabidopsis* accelerates flowering with abnormal floral organs in the first and second whorls (Xu et al., 2006).

*OMADS2* in the orchid *Oncidium* Gower Ramsey, a D-class gene, is specifically detected in stigmatic cavity and ovary (Hsu et al., 2010). This expression pattern is close to that of LMADS2 from *L. longiflorum*, which is exclusively present in the carpel (Tzeng et al., 2002). OMADS2 forms homodimers and heterodimers with OMADS4. Overexpression of *OMADS2 *in *Arabidopsis* leads to early flowering without any floral organ converstion (Hsu et al., 2010).

#### E-Class Genes

The members of the *SEP* MADS-box subfamily belong to the E-class genes that are necessary for the formation of all floral organs and floral meristem determinacy in *Arabidopsis*. The triple mutant in *SEP1/2/3/4* genes produce flowers with all floral organs converted to leaf-like organs (Pelaz et al., 2000; Ditta et al., 2004). *SEP* genes are present in angiosperms, but not gymnosperms, indicating that *SEP* genes may have been important for the existence of flowers (Nam et al., 2003). In *D. crumenatum*, the *SEP* ortholog *DcOSEP1* is detected in all floral organs, similarly to *Arabidopsis SEP*s. DcOSEP1 is able to interact with the DcOAP3A-DcOPI and DcOAP3B-DcOPI heterodimers, but not with DcOAP3A and DcOPI individually, indicating that DcOSEP1 is able to form a higher order protein complex with DcOAP3A-DcOPI or DcOAP3B-DcOPI, similar to their counterparts in *Arabidopsis* (Honma and Goto, 2001; Theissen and Saedler, 2001; Xu et al., 2006). In the *Dendrobium* Madame Thong-In orchid, *DOMADS1* and *DOMADS3* encode MADS-box proteins closely related with SEP1 and SEP3, respectively. *DOMADS1* is present in all the floral organs similarly to *DcOSEP1*, while *DOMADS3* is only present in the pedicel (Yu and Goh, 2000). In the *Oncidium* Gower Ramsey orchid, *OMADS6* (*OSEP3*) and *OMADS11* (*OSEP1*) encode MADS-box proteins homologous to *SEP3* and *SEP1/2*, respectively. Both genes are highly present in the sepal, petal, lip, and carpel, with weaker and undetectable expression in stamens for *OMADS6* and *OMADS11*, respectively (**Figure 3B**) (Chang et al., 2009). Overexpression of *OMADS6* in *Arabidopsis* leads to homeotic transformation of sepals into carpelliod structures and petals into stamen-like organs (Chang et al., 2009). In *P. equestris*, the four *SEP*-like *PeSEP* genes are expressed in flower buds with the expression of *PeSEP2* higher in floral stalk and column and *PeSEP3* in petals (Pan et al., 2014). Like the SEP proteins in *D. crumenatum*, PeSEP2, PeSEP3, and PeSEP4 proteins cannot interact with the B-class proteins PeMADS2, PeMADS4, PeMADS6, or the D-class protein PeMADS7 individually, but can form multimeric complexes with PeMADS2/6, PeMADS4/6 and PeMADS6/7. Only PeSEP1 is able to interact with PeMADS2, PeMADS4, PeMADS6, and PeMADS7 individually, and with PeMADS2/6, PeMADS4/6, and PeMADS6/7 (Pan et al., 2014). Silencing of *PeSEP3* by VIGS results in the conversion of tepal to leaf-like organ in *Phalaenopsis*, whereas silencing of *PeSEP2* does not greatly affect flower development (Pan et al., 2014), suggesting that these *PeSEPs* have divergent functions in orchid flower development.

The *AGL6*-like genes are similar to *SEP*-like genes and the *AGL6*-like gene in petunia functions like *SEP* genes in floral patterning (Rijpkema et al., 2009). It has been proposed to add *AGL6*-like genes to class-E genes. As described above, the *Arabidopsis AGL6* functions as a flowering promoter (Yoo et al., 2011). *Arabidopsis* has another *AGL6*-like gene called *AGL13*, which acts similarly to E-class *SEP* genes in specifying male and female gametophytes (Hsu et al., 2014). The orchid *Oncidium* Gower Ramsey also has two *AGL6*-like genes, *OMADS1* and *OMADS7*. *OMADS7* is expressed in all the floral organs, similar to that of E-class gene including *OMADS6* (**Figure 3B**) (Chang et al., 2009). *OMADS1* shows a different expression pattern which is in the lip and carpel, but not in other floral organs (**Figure 3B**) (Chang et al., 2009). Besides being early flowering, the flowers of *OMADS1* or *OMADS7* overexpression show homeotic transformation of sepals into carpel-like structures (Chang et al., 2009), indicating their dual roles in promoting floral transition and regulating floral organ formation. In *P. aphrodite*, the *AGL6*-like gene *PaAGL6-1* is expressed specifically in the lip, suggesting that *PaAGL6* may play an important role in lip formation (Su et al., 2013b). Subsequent studies have further revealed that the orchid *AGL6*-like genes play important roles in determining sepal/petal/lip formation (discussed in the following section). Together, *AGL6*-like genes may have diverse function in all four whorls of floral organs.

### The Orchid Perianth Code

Flowers in orchids and several other monocots such as lily, the sepals and petals are morphologically similar and are also collectively called tepals. This is different from *Arabidopsis* and other dicots flowers, in which sepals and petals have distinguished morphologies. To explain this difference in perianth organs specification, the modified ABC model has been proposed, in which the expression domain of B-class genes are extended to the outermost whorl of floral organs in many orchid species (**Figure 4B**) (Van Tunen et al., 1993; Bowman, 1997; Kramer et al., 2003; Mondragón-Palomino and Theißen, 2008). In the *Oncidium* Gower Ramsey orchid, the *AP3*-like gene *OMADS3* and the *PI*-like gene *OMADS8* are detected in all perianth organs (**Figure 3B**) (Chang et al., 2010).

However, the orchid flower has a median petal called lip, which has a highly diversified morphology and acts as the main attractor of pollinators. The specification of the lip cannot be simply explained by the modified “ABC model”. Several years of molecular studies of MADS-box proteins and orchid floral patterning have led to the discovery of the model of formation of perianth organs: the Perianth (P) code (**Figure 4C**) (Hsu et al., 2015). Based on this model, the two tetrameric MADS-box protein complexes, SP (sepal/petal) complex (OAP3-1/OAGL6-1/OAGL6-1/OPI) and L (lip) complex (OAP3-2/OAGL6-2/OAGL6-2/OPI), compete to promote the development of sepal/petal and lip, respectively. Different copies of B-class *AP3*-like genes (*OAP3-1* and *OAP3-2*) and *AGL6*-like genes (*OAGL6-1* and *OAGL6-2*) have different whorl-specific or whorl-biased expression patterns, providing the basis for the formation of SP and L complexes. Moreover, the relative levels of the two complexes may also determine the formation of various forms of intermediate lips or distinct lips in orchid.

## Concluding Remarks and Future Perspectives

The orchid family is the second largest family of angiosperms and has delighted cultivators for their unsurpassed beauty and complexity. The study on orchids has come a long way since people began gathering and propagating them under controlled environment. Recent findings in orchids, mainly *Cymbidium*, *Oncidium*, *Dendrobium*, and *Phalaenopsis*, have revealed that MADS-box proteins play critical roles in orchid flowering and floral patterning. The unraveled molecular mechanisms underlying orchid flowering and floral development can be applied to both classical orchid breeding and targeted manipulation of orchids for desired flowering traits and floral patterns. Orchids have many MADS-box genes, for example, 51 in *P. equestris* and 63 in *Dendrobium catenatum* (Cai et al., 2015; Zhang et al., 2016), however, only several of the MADS-box genes have been characterized, and most of them are shown be involved in orchid flowering or floral development. A recent study shows that MADS-box regulators might be relevant with the development of seeds without endosperm and epiphytism in orchids (Zhang et al., 2017), but revealing the functions of MADS-box genes in other develomental processes needs more future endeavor. In addition, in this review, the comparisons of orchid MADS-box genes to *Arabidopsis* have been included as many orchid genes are named and share similar functions to their closest orthologs in *Arabidopsis*. With the increasing number of MADS-box genes being studied in model monocots such as rice, wheat, barley, maize, and lily (Callens et al., 2018), more detailed comparisons of MADS-box gene functions between orchid and other monocots can be performed and discussed in the near future.

Nowadays, the function of orchid MADS-box genes, in many cases, is studied in heterologous plant systems (e.g. *Arabidopsis* and tobacco). In only several studies, the function of MADS-box genes has been examined by using transient overexpression/knockdown in orchids by VIGS or generating transgenic orchid overexpressing or knocking down of target genes (**Table 2**). For examples, silencing of *OAGL6-2* by VIGS has been done in *Oncidium* Gower Ramsey and *Phalaenopsis amabilis* hybrid to test the Perianth code, and *DOAP1* when overexpressed in *Dendrobium* Chao Praya Smile, leads to early flowering. To better understand the function of orchid genes, there is a need for more reliable and faster genetic transformation systems of different orchid species in orchid study and targeted orchid breeding with desired traits. Moreover, with modern genomic editing tools such as CRISPR-Cas9 (Clustered Regularly Interspaced Short Palindromic Repeats–Caspase 9), it is now possible to generate orchid mutants for *in vivo* functional characterizations (Kui et al., 2017). Indeed, CRISPR-Cas9 has been successfully used to create multiple mutants of MADS genes in the orchid *P. equestris* very recently (Tong et al., 2019).

With the advent of sequencing technologies, five orchid genomes have been released, including *Apostasia shenzhenica*, *D. catenatum*, *Dendrobium officinale*, *P. equestris*, and *Vanilla planifolia* (Cai et al., 2015; Yan et al., 2015; Zhang et al., 2016; Zhang et al., 2017; Hu et al., 2019). Moreover, the transcriptomes of several orchid species from different subfamilies are freely available in online databases, such as Orchidstra 2.0 (http://orchidstra2.abrc.sinica.edu.tw), OrchidBase 3.0 (http://orchidbase.itps.ncku.edu.tw) and OOGB (http://predictor.nchu.edu.tw/oogb) (Chang et al., 2011; Fu et al., 2011; Su et al., 2013a; Tsai et al., 2013; Chao et al., 2017; Tsai et al., 2017). Recently, the transcriptome of a Mediterranean orchid *Orchis italica* inflorescence have also been analyzed (Valoroso et al., 2019). Genome sequences and transcriptomic data have provided valuable information in aiding basic research and genomics-assisted horticultural breeding. The advent of molecular tools has allowed genomic analysis to determine the underlying mechanisms behind many morphological characteristics and developmental processes. With modern genomic editing tools available, it is now feasible to generate mutants or novel varieties in orchids. This would greatly help not only in the molecular genetic research of orchid biology, but also in generating novel orchid varieties with various desirable traits through targeted gene editing.

## Author Contributions

ZT and LS wrote the manuscript. WZ made the drawings of *Arabidopsis* and orchid plants. All authors read and approved of the manuscript.

## Funding

Preparation of the review has been supported by the intramural funding from Temasek Life Sciences Laboratory and National University of Singapore. 

## Conflict of Interest

The authors declare that the research was conducted in the absence of any commercial or financial relationships that could be construed as a potential conflict of interest.
